# Joint modelling compared with two stage methods for analysing longitudinal data and prospective outcomes: A simulation study of childhood growth and BP

**DOI:** 10.1177/0962280214548822

**Published:** 2016-07-11

**Authors:** A Sayers, J Heron, ADAC Smith, C Macdonald-Wallis, MS Gilthorpe, F Steele, K Tilling

**Affiliations:** 1 School of Social and Community Medicine, University of Bristol, Bristol, UK; 2 MRC Integrative Epidemiology Unit, University of Bristol, Bristol, UK; 3 Division of Epidemiology and Biostatistics, School of Medicine, University of Leeds, Leeds, UK; 4 Department of Statistics, London School of Economics, London, UK

**Keywords:** lifecourse epidemiology, joint model, multilevel model, measurement error, growth

## Abstract

There is a growing debate with regards to the appropriate methods of analysis of growth trajectories and their association with prospective dependent outcomes. Using the example of childhood growth and adult BP, we conducted an extensive simulation study to explore four two-stage and two joint modelling methods, and compared their bias and coverage in estimation of the (unconditional) association between birth length and later BP, and the association between growth rate and later BP (conditional on birth length). We show that the two-stage method of using multilevel models to estimate growth parameters and relating these to outcome gives unbiased estimates of the conditional associations between growth and outcome. Using simulations, we demonstrate that the simple methods resulted in bias in the presence of measurement error, as did the two-stage multilevel method when looking at the total (unconditional) association of birth length with outcome. The two joint modelling methods gave unbiased results, but using the re-inflated residuals led to undercoverage of the confidence intervals. We conclude that either joint modelling or the simpler two-stage multilevel approach can be used to estimate conditional associations between growth and later outcomes, but that only joint modelling is unbiased with nominal coverage for unconditional associations.

## 1 Introduction

Increasingly in epidemiology and medical research, there is interest in the relationship between both baseline level of, and change in, an exposure and a future outcome. Examples include changes in biomarkers in relation to disease incidence or progression (such as prostate specific antigen (PSA) in relation to progression of prostate cancer);^1^ changes in physiological variables in relation to disease outcomes (e.g. changes in renal function and subsequent arterial stiffness);^2^ and associations between physical and cognitive changes in later life.^3^ One change hypothesis that has been widely explored is the association between birth size, childhood growth and adult outcomes such as blood pressure (BP),^4–8^ and it is this example we focus on here – however, the methods are applicable to associations between linear change in any continuous exposure and a distal outcome.

**Figure 1.**
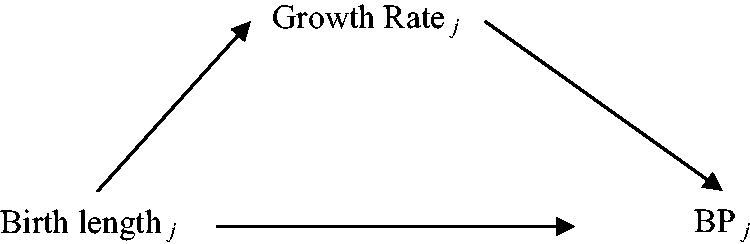
A schematic representation of causal associations between birth length, growth rate and BP.

We consider the example of a study aiming to investigate the association between birth length and linear growth rate during childhood on adult blood pressure (BP). We are interested in the total effect of birth length (B) on BP, and in the effect of linear growth rate (G) on BP, conditioning on birth length (Figure 1). Additional complexities that may occur in real data, such as non-linear growth, and an interaction between birth length and growth rate in their effect on the outcome are not considered. Studies relating birth length and growth to BP typically collect repeated data on height (length measured at birth and on at least one further occasion during childhood), and a later (adult) measure of BP. In our example, we assume that length is measured within 2 weeks of birth, and then height is measured at approximately 2.5, 5, 7.5 and 10 years of age.

A common approach is first to summarise the repeated measures of height, and then relate these summaries to the subsequent outcome. One simple method for carrying out the summary stage is to use the observed birth length and the latest childhood measure of height (or birth length and change between birth length and final height) as exposures;^9^ an alternative is to regress height on age within each individual to estimate their birth length and growth rate.^10^ More complex methods include using multilevel or other repeated measures methods to model the trajectories of height, and extracting summaries of these trajectories such as birth length and growth.^11,12^ Irrespective of the method used to summarise changes in exposure, linear regression models are then used to relate the summaries of change in height to the adult systolic BP, meaning that this stage can be carried out using standard statistical software. All the two-stage approaches share the potential problem that uncertainty in the estimates of birth length and growth are not taken into account in the confidence intervals for their associations with BP, meaning that standard errors may be underestimated.

This problem can be viewed in a measurement error framework, where the regression of blood pressure on birth height and growth is biased by the measurement error/intra-individual variation in height. Measurement error/intra-individual variation in the exposure (height) would tend to attenuate the coefficients between BP and height towards the null in the simple methods. It has been noted in the measurement-error literature that the two-stage multilevel method (also termed the regression calibration, or ‘RC’ method) provides consistent conditional effect estimates when the model relating exposures to outcome is linear,^13^ and that the individual regression method results in biased estimates of the conditional effects. However, more research is needed into the performance of all two-stage methods in estimating unconditional effects, and into their relative bias and coverage under different conditions.

In contrast to these two-stage approaches, the joint modelling approach aims to model birth length, growth and BP simultaneously, often using a bivariate growth model,^14^ or in a structural equation modelling framework.^15^ Joint modelling approaches are becoming more widely implemented in mainstream statistical software. However, joint models are more complex than the two-stage approaches outlined earlier, and it is not known whether any bias or under-coverage in the two-stage methods is large enough to warrant this extra modelling complexity.

In this simulation study, we compare the bias and coverage under different study conditions of six methods to estimate the association between birth length, linear growth and later BP. The two-stage methods we examine are: (1) a simple approach, which uses the observed birth length, and the difference between birth length and latest height measure (here, at approximately 10 years), divided by the difference between the ages at the two measurements, as an estimate of growth rate; (2) an individual regression (OLS) approach which estimates an individual’s birth length and growth rate from the parameters of the model regressing that individual’s height measures on age; (3) a multilevel growth model (MLM) where estimates of the individual random effects (shrunken residuals (or (4) inflated residuals) are used as estimates of birth length and growth rate. These are compared to two versions of a joint modelling approach: (5) a bivariate MLM, in which child growth and the adult outcome are modelled simultaneously and re-inflated residuals used to estimate the association between growth and outcome and (6) a structural equation model (SEM) which provides an alternative parameterisation of the bivariate model. In this study, we compare the bias and efficiency of using joint modelling and two-stage methods under different assumptions about the between- and within-individual variances of birth length and growth rate, and the relationship between them.

## 2 Methods

### 2.1 Simulation study design

We simulated a study where length is measured close to birth and at approximate ages of 2.5, 5, 7.5 and 10 years (*I* = 5 measurement occasions per individual). The exact ages (Age*_ij_*) at which height was measured for individual *j* at occasion *i* were drawn from a multivariate normal distribution with mean ages of 0, 2.5, 5, 7.5 and 10 years, and no covariance between occasions. The standard deviations of age at measurement at occasion *i* (σ_age_ *_i_*) were 0.5, with the exception of age 0 (σ_age__0_) which is 0.025 (approximately 95% of individuals had measurements within ± 2 weeks of zero). A single draw from the age distribution was used for all simulations, and age of measurement was checked to ensure that it was monotonically increasing across measurement occasions for each individual. The number of individuals in the study (*J*) was set to 1000, to illustrate a medium/large cohort study, and all individuals had all five measurements of height.

We simulated height for individual *j* at occasion *i* (H*_ij_*) from age for that individual at that occasion (Age*_ij_*) using a random intercept and random linear slope growth model

(1) $${\text{H}}_{\mathrm{ij}}=\beta_{0}{+\text{u}}_{0j}+(\beta_{1}{+\text{u}}_{1j}){\text{Age}}_{\mathrm{ij}}{\;+\text{e}}_{\mathrm{hij}}[{\text{u}}_{0j}{\text{u}}_{1j}]\sim N(0,\Omega_{u}):\Omega_{u}=[\sigma_{u0}^{2}\sigma_{u01}\sigma_{u1}^{2}][{\text{e}}_{\mathrm{hij}}]\sim \mathrm{iidN}(0,\sigma_{\mathrm{eh}}^{2})$$

Here, $\beta_{0}$ corresponds to the population average birth length, and $\beta_{1}$ to the population average linear growth rate. Random variables ${\text{u}}_{0j}$ and ${\text{u}}_{1j}$ correspond to the deviations from the population birth length and linear growth rate, respectively, for individual *j*, and ${\text{e}}_{\mathrm{hij}}$ represents the occasion level (level 1) residual for height for individual *j* at occasion *i*.

We then simulated a linear association between BP (${\text{BP}}_{j}$) and deviation from the population average birth length (${\text{u}}_{0j}$) and deviation from the population average growth rate (${\text{u}}_{1j}$) for individual *j*

(2) $${\text{BP}}_{j}=\gamma_{0}+\alpha_{3}{\text{u}}_{0j}+\alpha_{4}{\text{u}}_{1j}+{\text{e}}_{\text{BP}j}[{\text{e}}_{\text{BP}j}]\sim \mathrm{iidN}(0,\sigma_{e\text{BP}}^{2})$$

Here, $\gamma_{0}$ represents the population average systolic BP, $\alpha_{3}$ and $\alpha_{4}$ represent the effect of a 1 cm increase in birth length and a 1 cm y^−1^ increase in growth rate on systolic BP (mmHg) for individual *j*, conditional on both being included in the model.

### 2.2 Two-stage methods

Two-stage methods of analysis attempt to (1) summarise the growth process, i.e. birth length and growth rate, and the covariance between these (where the method considers this explicitly) and (2) use these summaries to investigate the associations of birth length and growth rate with the outcome of interest (BP). We begin by describing the second stage as this is common to all two-stage methods. We then outline the first stage, which differs for each approach.

The second stage starts by assuming that we have estimates of the birth length (${\overset{\wedge }{B}}_{j}$) and linear growth rate (${\overset{\wedge }{G}}_{j}$) of each individual *j* (*j = 1*, … , *J*) from the first stage. These are then related to the outcome of interest using two simple linear regression models. The first regression model assesses the total effect of the estimated birth length (${\overset{\wedge }{B}}_{j}$) of the jth individual (*j* = *1*, . . . , *J*) on their adult BP (BP*_j_*).

(3) $${\text{BP}}_{j}\;{=\alpha }_{0}+\alpha_{1}{\overset{\wedge }{B}}_{j}+{ɛ}_{1j}\;\text{and}\;{ɛ}_{1j}{\sim \text{N}(0,\sigma }_{ɛ1}^{2})$$

The second regression models the association between the estimated growth rate (${\overset{\wedge }{G}}_{j}$) and BP (BP*_j_*) conditional on birth length (${\overset{\wedge }{B}}_{j}$), where $\alpha_{4}$ is the parameter of interest. $\alpha_{3}$ is not of specific interest and cannot be directly interpreted given the potential of the reversal paradox.^16^

(4) $${\text{BP}}_{j}{=\alpha }_{2}{+\alpha }_{3}{\overset{\wedge }{\text{B}}}_{j}{+\alpha }_{4}{\overset{\wedge }{\text{G}}}_{j}+{ɛ}_{2j}\;\text{and}\;{ɛ}_{2j}{\sim \text{N}(0,\sigma }_{{ɛ}_{2}}^{2})$$

As the second stage of the analysis is estimated using OLS regression, the parameter estimates and their standard errors are obtained using standard methods.

### 2.3 The first stage

The first stage attempts to summarise the growth trajectory for individual *j* by estimating the true birth length (*B_j_*) and linear growth rate (*G_j_*) for each individual. The three two-stage methods considered here (the simple approach, the individual regression approach, and a multilevel model (MLM) for growth) differ in how they estimate birth length *B_j_* and growth rate *G_j_*.

#### 2.3.1 The simple approach

The simplest approach summarises the growth trajectory using the first observed measurement as an estimate of birth length, and the difference between the latest height measure (here, height at age of approximately 10 years) and birth length, divided by the elapsed time between measurements, as an estimate of growth rate. This idea is equivalent to that suggested by Lucas et al.,^8^ with the exception that they suggest adjusting for final height, which they recognise as a simple reparameterisation. This method assumes that the first observed height measure (H_1_*_j_*) is the best estimate of birth length. Justifiability of this assumption would depend on the timing of this first measurement. This simple method also assumes that the later measures of height provide no further information about birth length. Similarly, the estimate of growth rate is characterised by the difference between height at the first (H_1_*_j_*) and last measurement occasion (H_I_*_j_*), and assumes that intermediate measurements are uninformative.

(5) $${\overset{\wedge }{B}}_{j\text{SIMPLE}}={\text{H}}_{1j}$$

(6) $${\overset{\wedge }{G}}_{j\text{SIMPLE}}=\frac{({\text{H}}_{\text{I}j}-{\text{H}}_{1j})}{({\text{Age}}_{\text{I}j}-{\text{Age}}_{1j})}$$

If length/height is measured with error, then H_1_*_j_* is a composite of an individual’s ‘true’ birth length (*B_j_*) and measurement error e_1_*_j_*, i.e. H_1_*_j_* = *B_j_* + e_1_*_j_*. Therefore, unless there is no measurement error, the variance of ${\overset{\wedge }{B}}_{j\text{SIMPLE}}$ will be greater than the true variance of *B_j_*, and the relationship between BP and estimated birth length will be biased towards the null (see Appendix 1). The variance of ${\overset{\wedge }{G}}_{j\text{SIMPLE}}$ will also be greater than the true variance of *G_j_*. The correlation between ${\overset{\wedge }{B}}_{j\text{SIMPLE}}$ and ${\overset{\wedge }{G}}_{j\text{SIMPLE}}$ will be estimated as more negative than the true correlation between birth length and growth rate (i.e. if the true covariance is positive, then this method will underestimate the covariance between birth length and growth rate), due to mathematical coupling.^17,18^ However, the covariance between each of ${\overset{\wedge }{B}}_{j\text{SIMPLE}}$ and ${\overset{\wedge }{G}}_{j\text{SIMPLE}}$ and BP will be unbiased. Thus, whether the association between growth rate and BP (conditional on birth length) is overestimated or underestimated will depend on the relative sizes of the variances and covariance of birth length and growth rate.

#### 2.3.2 The individual regression (OLS) approach

An alternative approach is to fit *J* separate linear regression models (one for each individual) of height for individual *j* at time *i* (H*_ij_*) on age of that individual at each time-point (Age*_ij_*), via ordinary least squares (OLS).

(7) $$\exists j\in (1,J){:\text{H}}_{\mathrm{ij}}=\beta_{0j}+\beta_{1j}{\text{Age}}_{\mathrm{ij}}+{ɛ}_{j}\;\text{and}\;{ɛ}_{j}\sim {\text{N}(0,\sigma }_{{ɛ}_{3}}^{2})$$

The intercept and slope parameters of each individual regression are used to estimate birth length and growth rate for that individual

(8) $${\overset{\wedge }{B}}_{j\text{OLS}}=\beta_{0j},{\overset{\wedge }{G}}_{j\text{OLS}}=\beta_{1j}$$

${\overset{\wedge }{B}}_{j\text{OLS}}$ and ${\overset{\wedge }{G}}_{j\text{OLS}}$ are then used as estimates of birth length and growth rate in the second-stage models (3) and (4). Whilst the OLS method of regression will give unbiased estimates of *B_j_* and *G_j_* for individuals with fully observed measurements, the method fails to take into account the dependence between measurements, therefore violating the assumption of independence of ${ɛ}_{\mathrm{ij}}$, and underestimating the residual variance. As each individual trajectory is considered independently from the rest of the population, the between individual variance is overestimated, i.e. the variances of ${\overset{\wedge }{B}}_{j\text{OLS}}$ and ${\overset{\wedge }{G}}_{j\text{O}\mathrm{LS}}$ will be greater than the true variances of *B_j_* and *G_j_*. The sample covariance between estimated birth length (${\overset{\wedge }{B}}_{j\text{O}\mathrm{LS}}$) and growth rate (${\overset{\wedge }{G}}_{j\text{O}\mathrm{LS}}$) will be more negative than the true covariance (i.e. if the true covariance is positive, then this method will underestimate the covariance between birth length and growth rate). However, the covariance of the estimated birth length (${\overset{\wedge }{B}}_{j\text{O}\mathrm{LS}}$) and growth rate (${\overset{\wedge }{G}}_{j\text{O}\mathrm{LS}}$) with BP will be unbiased. Thus, as with the simple method, the association between birth length and BP will be biased towards the null, whereas that between growth rate and BP (conditional on birth length) could be biased in either direction.

#### 2.3.3 MLM approach

A further, more parsimonious, method would be to specify a MLM with random intercepts and slopes. This method simultaneously models the growth trajectories of all individuals, assuming that birth length and growth rate are normally distributed around the population mean birth length ($\beta_{0\text{MLM}}$) and growth rate ($\beta_{1\text{MLM}}$) with standard deviations σ_u0_, σ_u1_ and covariance σ_u01_.

(9) $${\text{H}}_{\mathrm{ij}}=\beta_{0j\text{MLM}}+\beta_{1j\text{MLM}}{\text{Age}}_{\mathrm{ij}}+{ɛ}_{\mathrm{ij}}\beta_{0j\text{MLM}}=\beta_{0\text{MLM}}{+\text{u}}_{0j}\beta_{1j\text{MLM}}=\beta_{1\text{MLM}}{+\text{u}}_{1j}[{\text{u}}_{0j}{\text{u}}_{1j}]\sim \text{N}({0,\Omega }_{\text{u}}){:\Omega }_{\text{u}}=[\sigma_{\text{u}0}^{2}\sigma_{\text{u}01}\sigma_{\text{u}1}^{2}][{ɛ}_{\mathrm{ij}}]\sim N(0,\sigma_{ɛ4}^{2})$$

The individual birth lengths and growth rates can be estimated by adding the level 2 shrunken residuals ${\overset{⌢}{u}}_{0j}$ and ${\overset{⌢}{u}}_{1j}$ (random effects estimates) to the estimates of the population mean birth length and growth rate, ${\overset{\wedge }{\beta }}_{0\text{MLM}}$ and ${\overset{\wedge }{\beta }}_{1\text{MLM}}$, to give the estimated birth length (${\overset{\wedge }{B}}_{j\text{MLM}}$) and growth rate (${\overset{\wedge }{G}}_{j\text{MLM}}$)

(10) $${\overset{\wedge }{B}}_{j\text{MLM}}={\overset{\wedge }{\beta }}_{0\text{MLM}}+{\overset{\wedge }{u}}_{0j}{\overset{\wedge }{G}}_{j\text{MLM}}={\overset{\wedge }{\beta }}_{1\text{MLM}}+{\overset{\wedge }{u}}_{1j}.$$

The individual level residuals ${\overset{⌢}{u}}_{0j}$ and ${\overset{⌢}{u}}_{1j}$ from the MLM above are the best linear unbiased predictors (BLUPS) for the individual random effects. They are, however, shrunk, in that they are a weighted average of the individual and the population average intercept and slope, with the weighting depending on the timing of measures, and the between- and within-individual variation (see Appendix 1). Thus, the sample distribution of the variances of the residuals will be smaller than the true variances of birth length and growth rate. The sample covariance between estimated birth length (${\overset{\wedge }{B}}_{j\text{MLM}}$) and growth rate (${\overset{\wedge }{G}}_{j\text{MLM}}$) will tend to be more positive than the true covariance (i.e. if the true covariance is positive, this method will overestimate it). In addition, the covariances between BP and birth length (${\overset{\wedge }{B}}_{j\text{MLM}}$) and growth rate (${\overset{\wedge }{G}}_{j\text{MLM}}$) will no longer be unbiased. However, we show that when both estimates (${\overset{\wedge }{B}}_{j\text{MLM}}$ and ${\overset{\wedge }{G}}_{j\text{MLM}}$) are included in the linear regression model for the outcome, the estimated associations between outcome and both intercept and slope are unbiased (see Appendix 1).

#### 2.3.4 MLM re-inflation approach

In order to mitigate the potential problem of under-estimation of the variances, shrunken residuals, ${\overset{⌢}{u}}_{0j}$ and ${\overset{⌢}{u}}_{1j}$, can be transformed (re-inflated) so that their sample variance and covariances reflect the model based estimates of $\Omega_{\text{u}}$. Re-inflated residuals are then added to the estimates of the population mean birth length and growth rate, ${\overset{\wedge }{\beta }}_{0\text{MLM}}$ and ${\overset{\wedge }{\beta }}_{1\text{MLM}}$, to give the estimated birth length (${\overset{\wedge }{B}}_{j\text{MLMINFL}}$) and growth rate (${\overset{\wedge }{G}}_{j\text{MLMINFL}}$) for each individual *j*. The process of re-inflation requires multiplying the estimated shrunken residuals by an upper triangular matrix of equal order to the number of random coefficients. A brief description of the process and an example of the code to perform the transformation is given in Appendix 1, and a more detailed description can be found in the original manuscript by Carpenter et al.^19^ The variances and covariances of estimated birth length (${\overset{\wedge }{B}}_{j\text{MLMINFL}}$) and growth rate (${\overset{\wedge }{G}}_{j\text{MLMINFL}}$) will be unbiased compared to the model based variances and covariances.

### 2.4 Joint modelling methods

#### 2.4.1 Bivariate MLM approach

In the bivariate model, the growth trajectory and adult BP models are estimated simultaneously.^14,20^ This model can be thought of as a combination of a two-level model for height, and a single level model for BP. The specification of the growth model is identical to the MLM presented previously. Additionally, a single level variance component model is estimated for BP and replaces the need to separately estimate equations (3) and (4).

(11) $${\text{H}}_{\mathrm{ij}}=\beta_{0j\text{BVM}}+\beta_{1j\text{BVM}}{\text{Age}}_{\mathrm{ij}}+{ɛ}_{\mathrm{ij}}\beta_{0j\text{BVM}}=\beta_{0\text{BVM}}{+\text{u}}_{0j}\beta_{1j\text{BVM}}=\beta_{1\text{BVM}}{+\text{u}}_{1j}{\text{BP}}_{j}=\beta_{2j\text{BVM}}\beta_{2j\text{BVM}}=\beta_{2\text{BVM}}{+\text{u}}_{2j}$$

The single level BP model estimates the population mean BP, and the residuals ${\overset{⌢}{u}}_{2j}$ estimate the individual deviation from that mean BP. As both models are estimated simultaneously, the variances and covariances of the residuals ${\overset{⌢}{u}}_{0j}$ (birth length), ${\overset{⌢}{u}}_{1j}$ (growth rate) and ${\overset{\wedge }{u}}_{2j}$ (BP) are jointly estimated, and Ω_u_ is now a 3 × 3 symmetric covariance matrix.

(12) $$[{\text{u}}_{0j}{\text{u}}_{1j}{\text{u}}_{2j}]\sim \text{N}({0,\Omega }_{\text{u}}){:\Omega }_{\text{u}}=[\sigma_{\text{u}0}^{2}\sigma_{\text{u}01}\sigma_{\text{u}1}^{2}\sigma_{\text{u}0\text{BP}}\sigma_{\text{u}1\text{BP}}\sigma_{\text{BP}}^{2}][{ɛ}_{\mathrm{ij}}]\sim N(0,\sigma_{ɛ5}^{2})$$

Using a similar method to that of the univariate MLM, the shrunken residuals ${\overset{⌢}{u}}_{0j}$, ${\overset{⌢}{u}}_{1j}$ and ${\overset{\wedge }{u}}_{2j}$ can be simultaneously re-inflated so that their variances and covariances reflect the true variance–covariance matrix. These inflated residuals can then be added to the population estimates of birth length (${\overset{\wedge }{\beta }}_{0\text{BVM}}$), growth rate (${\overset{\wedge }{\beta }}_{1\text{BVM}}$) and BP (${\overset{\wedge }{\beta }}_{2\text{BVM}}$) to give estimates of birth length (${\overset{\wedge }{B}}_{j\text{BVM}}$), growth rate (${\overset{\wedge }{G}}_{j\text{BVM}}$) and BP ($\overset{\wedge }{B}P_{j\text{BVM}}$) for each individual.

In a similar way to the two-stage approaches, but here using estimated instead of measured BP, these three estimates are then substituted into equations (3) and (4) and used to estimate the parameters of interest $\alpha_{1}$ and $\alpha_{4}$. Whilst deriving the parameters occurs in two stages, the growth and BP model is a single joint model. Thus, the variances and covariances of the estimated birth length, growth rate and BP will be unbiased estimates of the true variances and covariances, and the estimates of the parameters of interest $\alpha_{1}$ and $\alpha_{4}$ will be unbiased. However, this approach will not take into account the uncertainty in estimating the birth length, growth rate and BP from the bivariate model.

There are alternative methods which could be used to directly estimate $\alpha_{1}$ and $\alpha_{4}$ from the bivariate MLM. One method would be to manipulate the variances and covariances of Ω_u_, using a moment-based approach.^21^ In simple cases, this is fairly straightforward; however, calculating the corresponding standard errors is more complex. Simulation and moment-based methods have been developed,^22^ but these methods require normality assumptions to be made, and may only be appropriate in large samples.

In order to take into account the uncertainty in the residuals without relying on normality assumptions, a non-parametric bootstrap with replacement, with 1000 replicates, was used. The standard deviation of the 1000 bootstrap replicates was used as an estimate of the standard error. Normal theory confidence intervals were constructed using the observed point estimates and bootstrap standard errors, and percentile based confidence intervals were also calculated. This bootstrap estimation was only carried out for the baseline experimental scenario (see further in the text).

#### 2.4.2 Structural equation model

Using a structural equation modelling (SEM) framework, the bivariate outcome model can be reformulated, and framed in terms of measurement and structural models.

The measurement model (13) describes a model of linear dependence of the height H*_ij_* of an individual *j* at a given Age*_ij_* on two latent factors for birth length (B*_j_*_SEM_) and growth rate (G*_j_*_SEM_). The relationship is described by loadings λ_0*i*_ and λ_1*i*_ (where λ_0*i*_ = 1 and λ_1*i*_ = Age_*ij*_).

(13) $${\text{H}}_{\mathrm{ij}}=\lambda_{0i}B_{j\text{SEM}}+\lambda_{1i}G_{j\text{SEM}}+{ɛ}_{1\mathrm{ij}}$$

The structural model (14) directly relates the individual’s BP to the latent factors representing birth length and growth rate.

(14) $${\mathrm{BP}}_{j}=\alpha_{2}+\alpha_{3}B_{j\text{SEM}}+\alpha_{4}G_{j\text{SEM}}+{ɛ}_{2j}$$

Thus, the structural model has the same form as the model for BP in the two-stage approaches, (4), but with latent variables for birth length and growth replacing their sample estimates. One of the parameters of interest, the association between growth rate and BP conditional on birth length ($\alpha_{4}$), is estimated directly by this procedure. The other parameter of interest, the unconditional relationship between BP and birth length ($\alpha_{1}$) can be estimated by re-fitting the SEM but changing the structural model such that only birth length and BP are correlated

(15) $${\mathrm{BP}}_{j}=\alpha_{0}+\alpha_{1}B_{j\text{SEM}}+{ɛ}_{2j}$$

Alternatively, the unconditional relationship and standard error between BP and birth length ($\alpha_{1}$) can be estimated from the original SEM, where *r*_u01_ is the estimated correlation between the birth length and growth rate

(16) $$\alpha_{1}=[\alpha_{3}+\alpha_{4}(r_{\text{u}01}(\sigma_{\text{u}1}/\sigma_{\text{u}0}))]$$

For fixed measurement occasions (Age*_ij_* = Age*_i_*) and constraints on the loadings (λ_0_*_i_* = 1 and λ_1_*_i_* = Age*_i_*), this structural equation model is equivalent to the bivariate growth model described earlier. However, the structural equation model estimates the parameters of interest (and, importantly, their standard errors) directly, rather than requiring them to be derived from the model variance/covariance matrix.

### 2.5 Experimental scenarios

Five different experimental scenarios were chosen to explore the performance of different methods (in terms of bias and coverage) under different assumptions about the magnitude of variation in birth length, growth rate, correlation between birth length and growth rate, measurement error in the growth model and measurement error in the BP model. The number of replications for each of the scenarios outlined below was 1000.

Assuming a non-factorial design, standard deviations were set at σ_u0_ = 2.5, σ_u1_ = 0.5, σ_e_*_h_* = 2.0 and σ_eBP_* = *10, and correlation ρ_u01_ = 0.1. All other residual correlations were set equal to 0.

1. σ_u0_ (standard deviation of birth size, cm): 1.5, 2.0, (2.5), 3.0, 3.5.
2. σ_u1_ (standard deviation of growth rate, cm.y^−1^): 0.2, 0.3, 0.4, (0.5), 0.6, 0.7, 0.8, 0.9, 1.0.
3. σ*_e__hi_* (residual standard deviation in growth model, cm): 0.1, 1, (2), 3, 4, 5.
4. *ρ*_u01_ (correlation of birth size and growth rate): −0.6, −0.4, −0.2, −0.1, 0, (0.1), 0.2, 0.4, 0.6.
5. σ_eb_ (residual standard deviation in BP, mmHg): 8, 9, (10), 11, 12.

Values in parentheses are simulation defaults held constant in the other experimental scenarios, and the baseline experimental scenario held all parameters at these values.

Model parameters were fixed for all simulations at:

1. $\beta_{0}$(Birth Length) = 50
2. $\beta_{1}$(Growth Rate) = 9
3. $\alpha_{2}$(Mean BP) = 120
4. $\alpha_{3}$(Birth Length BP conditional association) = 0.5
5. $\alpha_{4}$(Growth Rate BP conditional association) = 2.0.

### 2.6 Summary statistics of interest

The statistics of primary interest are the estimated association between birth length and BP (${\overset{\wedge }{\alpha }}_{1}$) and the estimated association between growth rate and BP conditional on birth length (${\overset{\wedge }{\alpha }}_{4}$).

The expected unconditional association between birth length and BP is given by:

$\alpha_{1}=[\alpha_{3}+\alpha_{4}(\rho_{\text{u}01}(\sigma_{\text{u}1}/\sigma_{\text{u}0}))]$. For *k*th parameter (*k* = 1, 4), we investigate the relative bias of estimated coefficients $(({\overset{\wedge }{\alpha }}_{k}-\alpha_{k})/\alpha_{k})\cdot 100$. We also estimate coverage, where if $|{\overset{\wedge }{\alpha }}_{k}-\alpha_{k}|\leq 1.96\cdot \mathrm{SE}({\overset{\wedge }{\alpha }}_{k})$ the estimate was covered, and if $|{\overset{\wedge }{\alpha }}_{k}-\alpha_{k}|>1.96\cdot \mathrm{SE}({\overset{\wedge }{\alpha }}_{k})$ it was not.

### 2.7 Simulation implementation

The data were generated using Stata 12.1. The first stage of the simple and individualised regression approaches were also conducted in Stata 12.1,^23^ whereas the multilevel and bivariate MLMs were fitted in MLwIN 2.25^24^ via the runmlwin^25^ Stata command. The second stage of all two-stage methods was conducted in Stata 12.1. The structural equation model was fitted in Mplus^26^ via R using the MplusAutomation package.^27^ Full details of the syntax used to fit the models are listed in Appendix 1.

## 3 Results

**Figure 2.**
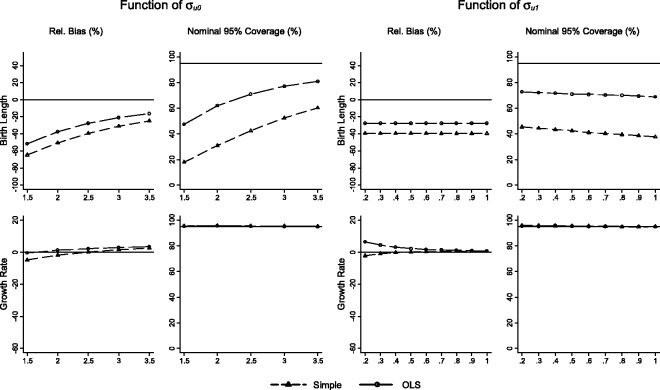
Performance of the simple and OLS methods displayed as relative bias, and nominal 95% coverage plotted as functions of σ_u0_ (standard deviation of birth length) and σ_u1_ (standard deviation of growth rate) for the effect of birth length on BP, and the effect of growth rate on BP conditional on birth length.

**Figure 3. fig3-0962280214548822:**
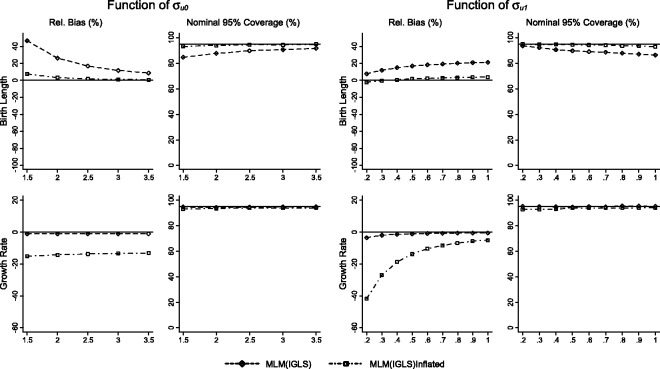
Performance of the multilevel model (MLM(IGLS)) and MLM with re-inflated residuals (MLM(IGLS) Inflated) methods displayed as relative bias, and nominal 95% coverage plotted as functions of σ_u0_ (standard deviation of birth length) and σ_u1_ (standard deviation of growth rate) for the effect of birth length on BP, and the effect of growth rate on BP conditional on birth length.

**Figure 4.**
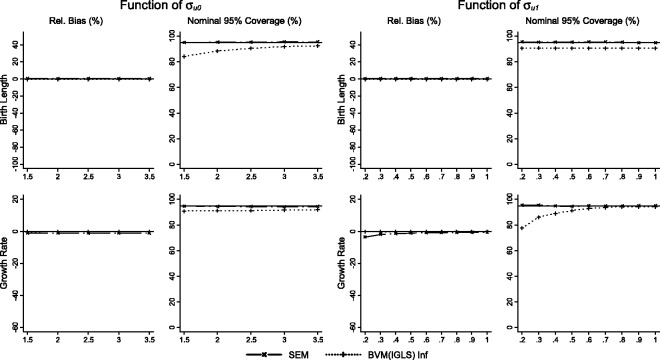
Performance of the structural equation model (SEM) and bivariate growth model with re-inflated residuals (BVM (IGLS) Inf) methods displayed as relative bias, and nominal 95% coverage plotted as functions of σ_u0_ (standard deviation of birth length) and σ_u1_ (standard deviation of growth rate) for the effect of birth length on BP, and the effect of growth rate on BP conditional on birth length.

Results for scenarios 1 (varying $\sigma_{\text{u}0}$) and 2 (varying $\sigma_{\text{u}1}$) for the simple and individualised growth trajectories, MLMs and joint models of growth and disease are presented in Figures 2–4, respectively. Each figure illustrates relative bias and nominal coverage plotted as a function of $\sigma_{\text{u}0}$ and $\sigma_{\text{u}1}$. The upper panels present bias and coverage for the association of birth length and BP, and the lower panels present bias and coverage for the association between growth rate and BP conditional on birth length.

If a model were to perfectly recover the estimates of interest, we would expect 0% relative bias which did not fluctuate with changes in the experimental values. Similarly, we would also expect nominal coverage to be approximately 95% across the range of experimental values used.

### 3.1 Two-stage simple and OLS methods

For the association between birth length and BP, both the simple and OLS methods are biased towards the null, and the simple method demonstrates more bias than the OLS method. The simple method has 18% nominal coverage rising to 60% as $\sigma_{\text{u}0}$ increases, and conversely falls from 45% to 37% as $\sigma_{\text{u}1}$ increases. Coverage using the OLS method is higher, rising steadily from 48% to 80% as $\sigma_{\text{u}0}$ increases; conversely, it falls from 72% to 67% as $\sigma_{\text{u}1}$ increases. The rise and fall in coverage is primarily a function of changing bias, as opposed to changes in confidence interval width.

The conditional growth rate/BP association shows less relative bias than the birth length/BP association for both methods. The simple method is modestly biased towards the null, with the bias reducing as $\sigma_{\text{u}0}$ and $\sigma_{\text{u}1}$ increase. The OLS method is biased away from the null with the size of the bias increasing with $\sigma_{\text{u}0}$ and decreasing with $\sigma_{\text{u}1}$. Coverage is greatly improved in comparison to the birth length/BP association for both the simple and OLS methods. Coverage of the simple and OLS method is approximately at expected levels in both scenarios, with modest improvements from 93% to 97% with increasing $\sigma_{\text{u}0}$.

A similar pattern of results (OLS more favourable than simple methods) can be seen for scenarios 3 (varying $\sigma_{\text{eh}}$), 4 (varying $\rho_{\text{u}01}$) and 5 (varying $\sigma_{\text{eBP}}$) (see Supplementary material, available at http://smm.sagepub.com/). For example, scenario 3 illustrates that simple estimates for birth length are consistently biased towards the null in comparison to OLS estimates. Both methods show little bias when $\sigma_{\text{eh}}$ is small; however, this increases as $\sigma_{\text{eh}}$ becomes larger. Estimates of birth length/BP association are unaffected by changes in $\rho_{\text{u}01}$ (scenario 4) and $\sigma_{\text{eBP}}$ (scenario 5), they are both consistently biased towards the null, the simple method more so than the OLS. Estimates of growth rate/BP associations (conditional on birth length) are biased towards the null when birth length and growth rate are negatively correlated, with that bias decreasing to zero and then increasing bias away from the null as birth length and growth rate are increasingly positively correlated. See Supplementary Figure 1 (see Supplementary material, available at http://smm.sagepub.com/).

### 3.2 Two-stage MLMs

Whilst the same MLM is estimated for both two-stage analyses, the difference in results between using shrunken or re-inflated residuals is substantial. For the association between birth length and BP, the use of shrunken residuals results in bias away from the null, with increasing bias as $\sigma_{\text{u}0}$ decreases or as $\sigma_{\text{u}1}$ increases. Whilst the coverage is modestly below expected levels, it increases from 85% to 93% as $\sigma_{\text{u}0}$ increases, and falls from 94% to 87% as $\sigma_{\text{u}1}$ increases. Re-inflating the residuals results in an attenuation of bias, and nominal coverage is at expected levels (95%) across the range of either $\sigma_{\text{u}0}$ or $\sigma_{\text{u}1}$.

The converse is demonstrated for the effect of growth rate. As expected, shrunken residuals are unbiased for changes in $\sigma_{\text{u}0}$ or $\sigma_{\text{u}1}$, and coverage is at expected levels (95%) across the ranges investigated. However, the re-inflation process caused an increase in bias towards the null (approximately 15%) across the range of $\sigma_{\text{u}0}$ investigated. The effect is more obvious in response to changes in $\sigma_{\text{u}1}$, where 40% bias is observed when $\sigma_{\text{u}1}$ is small, which slowly attenuates to 5% as $\sigma_{\text{u}1}$ increases. Coverage is only modestly below expected levels (93%) in both scenarios.

A similar pattern of results (inflation unbiased for birth length, shrunken residuals unbiased for growth rate) is seen for scenarios 3 ($\sigma_{\text{eh}}$), 4 ($\rho_{\text{u}01}$) and 5 ($\sigma_{\text{eBP}}$). The exception is for scenario 4, where inflated residuals yield biased results with respect to the birth length BP association when $\rho_{\text{u}01}$ is negative, see Supplementary Figure 2.

### 3.3 Joint models of growth and disease

Estimates from the bivariate MLM are unbiased (less than 1% relative bias) with respect to estimates of the birth length–BP association and the growth rate–BP association, as expected. The SEM method demonstrates unbiased results for the BP birth length association, and a small but persistent bias of 1% towards the null for the growth rate BP association in scenario 1, and a larger bias (3.5%) towards the null when $\sigma_{\text{u}1}$ is small which attenuates to less than 1% as $\sigma_{\text{u}1}$ increases. Nominal coverage of the SEM approach is at expected levels across all experimental scenarios, whereas nominal coverage using the two-stage approach of the bivariate growth model results in coverage slightly below expected levels. In the bivariate MLM, coverage for the association between birth length and BP ranges from 84% to 92% with increasing $\sigma_{\text{u}0}$, whereas it is approximately constant at 90% across all values of $\sigma_{\text{u}1}$. The pattern is nearly reversed with regard to the conditional association between growth rate and BP: changes in $\sigma_{\text{u}0}$ have little effect on nominal coverage of the bivariate MLM, which is approximately 91% across the range, whereas coverage is lower (78%) when $\sigma_{\text{u}1}$ is small which then steadily increases to near expected levels (94%) when $\sigma_{\text{u}1}$ becomes larger. Under-coverage is corrected, in the baseline scenario, using a non-parametric bootstrap. This approach resulted in nominal coverage using normal approximation confidence intervals (birth length 95.5%, growth rate 94.6%) or percentile confidence intervals (birth length 95.7%, growth rate 94.6%).

A similar pattern of results is seen for scenarios 3 ($\sigma_{\text{eh}}$), 4 ($\rho_{\text{u}01}$) and 5 ($\sigma_{\text{BP}}$). SEM methods show a small bias (1%) towards the null and nominal coverage is at expected levels for all scenarios. The bivariate growth model two-stage method is unbiased, but nominal coverage falls with increasing $\sigma_{\text{eh}}$, and also falls with increasing $\rho_{\text{u}01}$ correlation, see Supplementary Figure 3.

## 4 Discussion

We have shown algebraically that the two-stage process using a MLM to estimate growth parameters and then relating these to the distal outcome in a second stage will give unbiased estimates of the conditional associations between both growth parameters and outcome. Our simulations confirmed this, and also showed that using the same process to estimate the unconditional association between birth length and outcome leads to bias. We have shown that the two-stage bivariate MLM (with re-inflation) is unbiased in all the scenarios investigated, although with under-coverage of confidence intervals. We have also demonstrated that SEM produces a small bias towards the null in the estimation of the association between growth rate and BP (due to the different distribution of individual ages at birth compared to the other time-points). The simple and OLS two-stage methods result in biased estimates of the association between BP and birth length, and less biased estimates of the association between BP and growth rate, conditional on birth length.

The simple two-stage method illustrated substantial bias in the presence of measurement error, and underperformed in comparison to the OLS method with regards to estimates of the effect of birth length on BP. The OLS method demonstrated considerable bias with regards to the estimates of birth length on BP and nominal coverage was also poor. The MLM two-stage approach with inflated residuals demonstrated the least bias with regards to the association of birth length with BP, and considerably outperformed the use of shrunken residuals, which was nearly as biased (although in the opposite direction) as the OLS method. Therefore, the two-stage method of choice when investigating the effects of birth length on BP would be the MLM with inflated residuals. However, the process of re-inflating residuals is not unique, and the transformation does not necessarily preserve the relationship between the empirical residual and the outcome of interest. Using the lower triangular matrices of the Cholesky decomposition during re-inflation results in inflated residuals for birth length which are a simple linear transformation of the uninflated residuals. However, using the upper triangular Cholesky decomposition results in inflated residuals for birth length which are a linear combination of the uninflated residuals for growth rate and those for birth length and so does not result in unbiased associations.

In terms of the association of BP with growth rate (conditional on birth length), the most biased two-stage method was the MLM with inflated residuals, which biases results towards the null. The bias occurs because the association between growth rate and BP is distorted during the inflation process, which is a complex transformation that depends on the shrunken residuals of both birth length and growth rate. The simple method also illustrates biases towards the null, but to a lesser extent than those with inflated residuals. The OLS method illustrates biases worse than the simple method under some circumstances, despite the intuitive incorporation of all relevant data. However, the two-stage method using the MLM with shrunken residuals led to unbiased results, since the consistent shrinkage of both birth length and growth rate residuals preserve the association between BP and growth rate conditional on birth length. Nominal coverage is preserved at expected levels by the inflated standard errors due to the reduced residual variance.

The bivariate growth model, which simultaneously generates growth and BP residuals, which are in turn re-inflated, leads to unbiased results in all scenarios. However, this method results in under-coverage of 5% in scenarios 1 and 2. The unbiased result and less than optimal coverage needs to be balanced against the minor bias yielded by SEM method (in this example, due to unbalanced data), and the full propagation of uncertainty and correct 95% nominal coverage, or the computationally intensive nature of the non-parametric bootstrap, which fully incorporates the uncertainty from the growth model.

Approaches for tackling this problem have been suggested, in the context of the measurement error. It has been noted that using parameters from a linear MLM (the ‘RC’ method) results in unbiased conditional effect estimates when the model relating exposures to outcome is linear.^13^ The same paper noted that the individual regression method resulted in biased estimates of the conditional effects. However, for non-linear models for the outcome (e.g. a logistic model for a binary distal outcome), this method remains biased (although with reduced bias compared to the individual regression method), and alternatives have been proposed.^28^ Given the difficulties in estimating joint models with a binary outcome, more research is needed into the size of the bias when using the two-stage multilevel approach in the non-linear case, and the ease of implementation of alternatives.

## 5 Future work

Whilst this simulation study highlights how variation in specific parts of the data-generating process affect the estimation of the effect of either birth length or growth rate on BP, we have not explored a full factorial experimental design, therefore combinations of unfavourable scenarios may result in unacceptable bias and poor coverage. Additionally, we have only explored these effects when the number of observations is the same in each individual, and not explored the consequences of imbalance and missing data for the simple and OLS methods. We briefly explored the consequences of equalising the size of the effects between growth parameters and BP, and found similar associations, i.e. biases in birth length and BP association were greater than biases in the growth rate and BP associations (results available from authors upon request). Furthermore, we did not vary the direction and magnitude of the association between growth parameters and BP and therefore we are unable to explore the potential reversal paradox described by Tu and colleagues.^16^ Similarly, we did not investigate the effect of population size or frequency of measurement in relation to the observed biases, and the stability of the six methods with small numbers of individuals and/or infrequent measurements may be different to that seen here.

We did not examine the effect of violating model assumptions. In particular, whilst growth (and change in other anthropometric variables) is often linear over short periods of time, non-linear models will generally be required for examining change over longer periods. More complex data-generating processes could also be considered, for example by creating an interaction between birth length and growth rate in their association with the outcome. SEM or path analyses could be used to examine the mediation of the association between birth length and BP by growth rate.

## 6 Conclusions

The joint modelling approach which takes into account and incorporates the variation of growth process into the estimation of effects on subsequent outcomes is clearly the preferred method, giving unbiased estimates of both the conditional and unconditional associations of birth length and growth with BP. Given the requirement for specialist software and the greater technical difficulty in fitting the joint models, this option may not be viable for all researchers without specialist training. An alternative would be to use the two-stage MLM approach to estimate conditional associations, where an experienced analyst can derive the residuals, and less-experienced researchers can use them as exposures in standard regression models. Where measurement error in the repeated outcome is low, this approach may result in little bias even for unconditional associations.

This simulation study could change the interpretation of previously reported null findings, as many of the methods commonly used result in biases towards the null and poor nominal coverage. Thus, reanalysis with more suitable methods may reduce both the type I error rate and the heterogeneity in the current literature.[Fig fig3-0962280214548822]

## Supplementary Material

Supplementary material

Supplementary material
