# Supplementary material for: Joint modelling compared with two stage methods for analysing longitudinal data and prospective outcomes: A simulation study of childhood growth and BP
Source: Stat Methods Med Res. 2016 Jul 11;26(1):437–52. doi: 10.1177/0962280214548822 (PMC5476230; doi:10.1177/0962280214548822)
Supplement: Supplementary material [file SMM548822_appendix.pdf]

Appendix

1. Proof that two-stage process using a multilevel model is unbiased

We consider the general case of a two-stage process that incorporates a multilevel model in the first stage. Having proved the result in the general case we will apply it to the specific example described in the main body of the paper. Therefore, let the multilevel model

$$Y_j = X_j\beta + Z_j u_j + \varepsilon_j$$

be the first stage, in which Y_j is a vector of responses for subject j, the vector β and matrix X_j are the global fixed effects and corresponding design matrix for subject j, the vector u_j contains the random effects for subject j, which parametrizes that individuals growth trajectory, with corresponding design matrix Z_j. Assume that $u_j \sim N(0, \Omega)$ and $\varepsilon_j \sim N(0, \sigma^2 I)$ are independent of each other and independent between subjects. The second stage is given by the linear model

$$W_j = a + u_j^T \alpha + \zeta_j,$$

in which W_j is the outcome of interest for subject j and $\zeta_j \sim N(0, s^2)$.

Letting

$$Y = \begin{pmatrix} Y_1 \\ \vdots \\ Y_J \end{pmatrix},\ X = \begin{pmatrix} X_1 \\ \vdots \\ X_J \end{pmatrix},\ Z = \begin{pmatrix} Z_1 & & \\ & \ddots & \\ & & Z_n \end{pmatrix},\ u = \begin{pmatrix} u_1 \\ \vdots \\ u_J \end{pmatrix},\ \varepsilon = \begin{pmatrix} \varepsilon_1 \\ \vdots \\ \varepsilon_J \end{pmatrix},$$
$$G = \begin{pmatrix} \Omega & & \\ & \ddots & \\ & & \Omega \end{pmatrix},\ W = \begin{pmatrix} W_1 \\ \vdots \\ W_J \end{pmatrix},\ A = \begin{pmatrix} \alpha^T & & \\ & \ddots & \\ & & \alpha^T \end{pmatrix},\ \zeta = \begin{pmatrix} \zeta_1 \\ \vdots \\ \zeta_J \end{pmatrix},$$

and $\mathbf{1}$ denote a vector of dimension *J* (where *J*=the total number of individuals) consisting only of 1s, we can write the first stage model as

$$Y = X\beta + Zu + \varepsilon,$$

where $u \sim N(0, G)$ and $\varepsilon \sim N(0, \sigma^2 I)$, and the second stage model as

$$W = a\mathbf{1} + Au + \zeta,$$

where $\zeta \sim N(0, s^2 I)$. In the two-stage approach, we first estimate the BLUPs from the multilevel model in the first stage. These are known to be $\hat{u} = GZ^T V^{-1}(Y - X\hat{\beta})$, where $\hat{\beta} = (X^T V^{-1} X)^{-1} X^T V^{-1} Y$ and $V = ZGZ^T + \sigma^2$. We then estimate a and α using the least-squares estimate $(\hat{U}^T \hat{U})^{-1} \hat{U}^T W$, where

$$\hat{U} = \begin{pmatrix} 1 & \hat{u}_1^T \\ \vdots & \vdots \\ 1 & \hat{u}_J^T \end{pmatrix}.$$

To find the bias of this estimator, first consider $E(W|\hat{u})$. We can write $\hat{u} = GZ^T V^{-1} HY$, where $H = I - X(X^T V^{-1} X)^{-1} X^T V^{-1}$. Note that H is idempotent since $H^2 = H$, that $V^{-1}H = H^T V^{-1}$, and that $HX\beta = 0$ and hence $HY = H(Zu + \varepsilon)$.

The joint distribution of W and $\hat{u}$ is multivariate normal. They are related through the expression

$$\begin{pmatrix} W \\ \hat{u} \end{pmatrix} = \begin{pmatrix} a\mathbf{1} & A & I & 0 \\ 0 & GZ^TV^{-1}HZ & 0 & GZ^TV^{-1}H \end{pmatrix} \begin{pmatrix} 1 \\ u \\ \zeta \\ \varepsilon \end{pmatrix},$$

from which we can deduce their expectations

$$E\begin{pmatrix} W \\ \hat{u} \end{pmatrix} = \begin{pmatrix} a\mathbf{1} \\ 0 \end{pmatrix},$$

and covariance

$$var\begin{pmatrix} W \\ \hat{u} \end{pmatrix} = \begin{pmatrix} AGA^T + s^2 & AGZ^TH^TV^{-1}ZG \\ GZ^TV^{-1}H^TZGA^T & GZ^TH^TV^{-1}ZG \end{pmatrix}.$$

To simplify $var(\hat{u})$ we have used the above facts about H. Therefore

$$\begin{aligned} E(W|\hat{u}) &= E(W) + cov(W,\hat{u})var(\hat{u})^{-1}\big(\hat{u} - E(\hat{u})\big) \\ &= a\mathbf{1} + AGZ^TH^TV^{-1}ZG(GZ^TH^TV^{-1}ZG)^{-1}(\hat{u} - 0) \\ &= a\mathbf{1} + A\hat{u} \end{aligned}$$

and hence

$$E(W|\hat{u}) = \hat{U}\begin{pmatrix} a \\ \alpha \end{pmatrix}.$$

Finally we show that $\left(\hat{U}^T\hat{U}\right)^{-1}\hat{U}^TW$ is an unbiased estimator of a and α, because

$$\begin{aligned} E\left(\left(\hat{U}^T\hat{U}\right)^{-1}\hat{U}^TW\right) &= E\left(E\left(\left(\hat{U}^T\hat{U}\right)^{-1}\hat{U}^TW\middle|\hat{u}\right)\right) \\ &= E\left(\left(\hat{U}^T\hat{U}\right)^{-1}\hat{U}^TE(W|\hat{u})\right) \\ &= E\left(\left(\hat{U}^T\hat{U}\right)^{-1}\hat{U}^T\hat{U}\begin{pmatrix} a \\ \alpha \end{pmatrix}\right) \\ &= E\begin{pmatrix} a \\ \alpha \end{pmatrix} = \begin{pmatrix} a \\ \alpha \end{pmatrix}. \end{aligned}$$

If β and u are of the same dimension, and we choose to use the estimates $\hat{\beta} + \hat{u}_j$ instead of $\hat{u}_j$ in the second stage, then the estimate in the second stage is $\left(\hat{F}^T\hat{F}\right)^{-1}\hat{F}^TW$, where

$$\hat{F} = \begin{pmatrix} 1 & \beta^T + \hat{u}_1^T \\ \vdots & \vdots \\ 1 & \beta^T + \hat{u}_J^T \end{pmatrix}.$$

In this case we have

$$E(W|\hat{u}) = a\mathbf{1} + A\hat{u} = a\mathbf{1} - AD\hat{\beta} + A\big(D\hat{\beta} + \hat{u}\big) = \hat{F}\begin{pmatrix} a - \hat{\beta}^T\alpha \\ \alpha \end{pmatrix},$$

where

$$D\hat{\beta} = \begin{pmatrix} \hat{\beta} \\ \vdots \\ \hat{\beta} \end{pmatrix}.$$

Hence

$$\begin{aligned}
E\left(\left(\hat{F}^T\hat{F}\right)^{-1}\hat{F}^T W\right) &= E\left(E\left(E\left(\left(\hat{F}^T\hat{F}\right)^{-1}\hat{F}^T W \middle| \hat{u}\right) \middle| \hat{\beta}\right)\right) \\
&= E\left(E\left(\left(\hat{F}^T\hat{F}\right)^{-1}\hat{F}^T E(W|\hat{u}) \middle| \hat{\beta}\right)\right) \\
&= E\left(E\left(\left(\hat{F}^T\hat{F}\right)^{-1}\hat{F}^T\hat{F}\begin{pmatrix} a - \hat{\beta}^T\alpha \\ \alpha \end{pmatrix} \middle| \hat{\beta}\right)\right) \\
&= E\left(\begin{pmatrix} a - \hat{\beta}^T\alpha \\ \alpha \end{pmatrix} \middle| \hat{\beta}\right) = E\begin{pmatrix} a - \hat{\beta}^T\alpha \\ \alpha \end{pmatrix} = \begin{pmatrix} a - \beta^T\alpha \\ \alpha \end{pmatrix}.
\end{aligned}$$

The estimate in the second stage is unbiased for α.

In the specific example of the main body of the paper, we have

$$Y_j = \begin{pmatrix} \mathrm{H}_{1j} \\ \vdots \\ \mathrm{H}_{Ij} \end{pmatrix} \quad X_j = Z_j = \begin{pmatrix} 1 & \mathrm{Age}_{1j} \\ \vdots & \vdots \\ 1 & \mathrm{Age}_{Ij} \end{pmatrix} \quad \beta = \begin{pmatrix} \beta_0 \\ \beta_1 \end{pmatrix} \quad u_j = \begin{pmatrix} u_{0j} \\ u_{1j} \end{pmatrix} \quad \varepsilon_j = \begin{pmatrix} e_{h1j} \\ \vdots \\ e_{hIj} \end{pmatrix}$$

$$W_j = \mathrm{BP}_j \quad a = \alpha_2 \quad \alpha = \begin{pmatrix} \alpha_3 \\ \alpha_4 \end{pmatrix} \quad \zeta_j = e_{bj}$$

$$\Omega = \Omega_{\mathrm{u}} \quad \sigma^2 = \sigma_{eh}^2$$

$$s^2 = \sigma_{eb}^2$$

2. Residual Reinflation,

The process of reinflating shrunken residuals requires finding a transformation so that

$$\hat{U}^* = \hat{U}A$$

Where $\hat{U}^*$ is a matrix of the inflated residuals indexed by j rows, $\hat{U}$ is a matrix of the shrunken residuals indexed by j rows and A is a matrix of equal order Ω_{u}.

Matrix A is found by calculating the Cholesky decompositions of the empirical covariance of estimated residuals and the maximum likelihood estimate of the random effects covariance matrix.

The empirical covariance matrix is calculated so that

$$S = \hat{U}^T\hat{U} / J$$

and the corresponding maximum likelihood estimate of the random effects covariance matrix is

$$R = \Omega_u$$

R and *S* are rewritten in terms of lower triangular Cholesky decompositions

$$R = L_R L_R^T$$

$$S = L_S L_S^T$$

Then A (an upper triangular matrix) is calculated by choosing the lower triangular matrices of R and S.

$$A = \left(L_R L_S^{-1}\right)^T$$

3. Model syntax

Simple Approach (Stata Syntax)

```
// Start with data saved in long format
//sort id time
        sort id time
// Generate estimate of birth length
        gen bl_simple = ht if time==1
// Generate growth rate estimate
        gen gr_simple = (ht[_n+4] - ht[_n]) / (age[_n+4] -age[_n] ) if time==1
// Second stage analysis
        reg bp bl_simple if time==1
        reg bp bl_simple gr_simple if time==1
```

Individual trajectories approach (Stata Syntax)

```
// Start with data saved in long format
// Create a consecutive id
        egen new_id = group(id)
// Generate empty birth length and growth rate variables
        gen bl_ols = .
        gen gr_ols = .
// Summarise the id to obtain lowest and highest id
        sum new_id
// Start loop
        forvalues id = `r(min)'(1)`r(max)' {
// Do individual regression
        reg ht age if id==`id'
// Replace birth length and growth rate with individual estimates
        replace bl_ols = _b[_cons] if id==`id' & time==1
        replace gr_ols= _b[age] if id==`id' & time==1
                }
// Second stage analysis
reg bp bl_ols
reg bp bl_ols gr_ols
```

Multi-level model approach (Stata Syntax)

```
// Start with data saved in long format
// Fit random intercept slope model using runmlwin
runmlwin ht cons age , level1(time: cons) level2(id: cons age , residuals(mlm_res, )) igls
// Rename the residuals
rename mlm_res0 bl_mlm
rename mlm_res1 gr_mlm
// Second stage analysis
reg bp      bl_mlm if time==1
reg bp      bl_mlm gr_mlm if time==1
```

Multi-level model inflated residual approach (Stata Syntax)

```
// Start with data saved in long format
// Fit random intercept slope model using runmlwin
        runmlwin ht cons age , level1(time: cons) level2(id: cons age , residuals(mlm_res, ))
// Reshape data into wide format
        reshape wide age ht e  , i(id) j(time)
// Add Omega U matrix
        matrix R =e(RP2)
// Add estimated residuals to a matrix
        matrix N =e(N_g)
        local J= N[1,1]
        set matsize `J'
        mkmat  mlm_res0 mlm_res1 , matrix(Uhat)
// Calculate the empirical sampling covariance matrix
        matrix S = Uhat'*Uhat / `J'
// Do The Cholesky Decomposition
        matrix Ls = cholesky(S)
        matrix Lr = cholesky(R)
```

```
//  Create Matrix A
        matrix A = (Lr* inv(Ls))'
//  Create U*
        matrix Ustar = Uhat*A
// Create Inflated Residuals
        svmat Ustar , names(Ustar_)
// Rename the inflated residuals
        rename Ustar_1 bl_mlm_inf
        rename Ustar_2 gr_mlm_inf
// Second stage analysis
        reg bp      bl_mlm_inf
        reg bp      bl_mlm_inf gr_mlm_inf
```

Bivariate growth model inflated residual approach (Stata Syntax)

```
// Start with data saved in wide format
// Reshape data into bivariate format
        reshape wide age ht e  , i(id) j(time)
        rename bp bp6
        reshape long age bp ht e , i(id) j(time_1)
// Fit a bivariate growth model
        runmlwin (ht cons age, eq(1)) (bp cons, eq(2)) , ///
        level1(time:   (cons, eq(1)) ) ///
        level2(id: (cons age, eq(1)) (cons , eq(2)) , residuals( bvgm_res , norecode) )
// Reshape data into wide format
        reshape wide age bp ht e  , i(id) j(time)
// Add Omega U matrix
        matrix R =e(RP2)
// Add estimated residuals to a matrix
        matrix N =e(N_g)
        local J= N[1,1]
        set matsize  `J'
        mkmat   bvgm_res0 bvgm_res1 bvgm_res2 , matrix(Uhat)
// Calculate the empirical sampling covariance matrix
        matrix S = Uhat'*Uhat / `J'
// Do The Cholesky Decomposition
        matrix Ls = cholesky(S)
        matrix Lr = cholesky(R)
//  Create Matrix A
        matrix A = (Lr* inv(Ls))'
//  Create U*
        matrix Ustar = Uhat*A
// Create Inflated Residuals
        svmat Ustar , names(Ustar_)
// Rename the inflated residuals
        rename Ustar_1 bl_bvgm_inf
        rename Ustar_2 gr_bvgm_inf
        rename Ustar_3 bp_bvgm_inf
// Calculate the effects of interest
        reg bp_bvgm_inf bl_bvgm_inf
        reg bp_bvgm_inf bl_bvgm_inf gr_bvgm_inf
```

Non-parametric bootstrap bivariate growth model inflated residual approach (Stata Syntax)

```
// Start with data saved in wide format
// Prepare bootstrap data storage
        cap postclose bootstrap
        postfile bootstrap i birth_length growth_rate using myboot.dta , replace

// Start bootstrap loop
        forvalues bs_i = 1/10 {
// Read in data
        use  growthdata.dta , clear
        gen  cons=1
// Randomly sample data with replacement
        bsample , cluster(id)
// Generate a bootstrap id
        gen bs_id=_n
// Reshape into a bivariate format
        rename bp bp6
        reshape long age bp ht e , i(bs_id) j(time_1)
```

```
// Fit a bivariate growth model
	runmlwin (ht cons age, eq(1)) (bp cons, eq(2)) , ///
	level1(time:   (cons, eq(1)) ) ///
	level2(bs_id: (cons age, eq(1)) (cons , eq(2)) , residuals( bvgm_res , norecode) ) /// nopause
// Residual reinflation
// Reshape into a wide format
reshape wide age bp ht e  , i(bs_id) j(time)

// Add Omega U matrix
	matrix R =e(RP2)
// Add estimated residuals to a matrix
	matrix N =e(N_g)
	local J= N[1,1]
	set matsize  `J'
	mkmat   bvgm_res0 bvgm_res1 bvgm_res2 , matrix(Uhat)
// Calculate the empirical sampling covariance matrix
	matrix S = Uhat'*Uhat / `J'
// Do The Cholesky Decomposition
	matrix Ls = cholesky(S)
	matrix Lr = cholesky(R)
//  Create Matrix A
	matrix A = (Lr* inv(Ls))'
//  Create U*
	matrix Ustar = Uhat*A
// Create Inflated Residuals
	svmat Ustar , names(Ustar_)
	rename Ustar_1 bl_mlm_inf
	rename Ustar_2 gr_mlm_inf
	rename Ustar_3 bp_mlm_inf
// Calculate the effects of interest
	reg bp_mlm_inf bl_mlm_inf
	local birth_length = _b[bl_mlm_inf]
	reg bp_mlm_inf bl_mlm_inf gr_mlm_inf
	local growth_rate = _b[gr_mlm_inf]
// Save out results
	post bootstrap (`bs_i') (`birth_length') (`growth_rate')
// End bootstrap loop
	}
// close storage
	postclose bootstrap
// Read in bootstrap results
	use my_boot.dta , clear
// Means and SE
	su birth_length  growth_rate
// Percentile confidence intervals
	centile birth_length  growth_rate , c(2.5 97.5)
```

SEM joint model of growth and BP (Mplus syntax)

```
TITLE: SEM Joint model of growth and BP
DATA: FILE IS "growthdata.txt";
VARIABLE: NAMES ARE id age1-age5 ht1-ht5 bp;
USEVARIABLES = ht1-ht5 age1-age5 bp;
TSCORES = age1-age5;
ANALYSIS: TYPE = RANDOM;
   PROC = 2(starts);
MODEL:
   i s | ht1-ht5 AT age1-age5;
   [i s];
   i (intvar);
   s (slopevar);
   i WITH s (cov);
   bp ON i (a1);
   bp ON s (a2);
   ht1-ht5 (1);
model constraint:
   new(corr_u01 crude_bl crude_sl sd_u0 sd_u1);
   sd_u0 = intvar**(0.5);
   sd_u1 = slopevar**(0.5);
   corr_u01 = cov/((sd_u0)*(sd_u1));
   birth_length = a1+ (a2 * (corr_u01 * sd_u1/sd_u0));
   growth rate = a2 ;
```
