# Supplementary material for: Joint modelling compared with two stage methods for analysing longitudinal data and prospective outcomes: A simulation study of childhood growth and BP
Source: Stat Methods Med Res. 2016 Jul 11;26(1):437–52. doi: 10.1177/0962280214548822 (PMC5476230; doi:10.1177/0962280214548822)

Supplementary Figure 1:

Model performance of the simple and OLS method are displayed as relative bias, and nominal 95% coverage plotted as functions of σ_{eh} (standard deviation of measurement error at each data collection), ρ_{u01} (correlation between birth length and growth rate), and of σ_{eBP} (standard deviation of growth model and BP association) for the effect of birth length on BP, and the effect of growth rate on BP conditional on birth length.

Supplementary Figure 2:

Model performance of the multilevel model (MLM(IGLS)) and multilevel model with re-inflated residuals (MLM(IGLS) Inflated) method are displayed as relative bias, and nominal 95% coverage plotted as functions of σ_{eh} (standard deviation of measurement error at each data collection), ρ_{u01} (correlation between birth length and growth rate), and of σ_{eBP} (standard deviation of growth model and BP association) for the effect of birth length on BP, and the effect of growth rate on BP conditional on birth length.

Supplementary Figure 3:

Model performance of the structural equation model (SEM) and bivariate growth model with re-inflated residuals (BVM (IGLS) Inf.) method are displayed as relative bias, and nominal 95% coverage plotted as functions of σ_{eh} (standard deviation of measurement error at each data collection), ρ_{u01} (correlation between birth length and growth rate), and of σ_{eBP} (standard deviation of growth model and BP association) for the effect of birth length on BP, and the effect of growth rate on BP conditional on birth length.

Supplementary Figure 1:

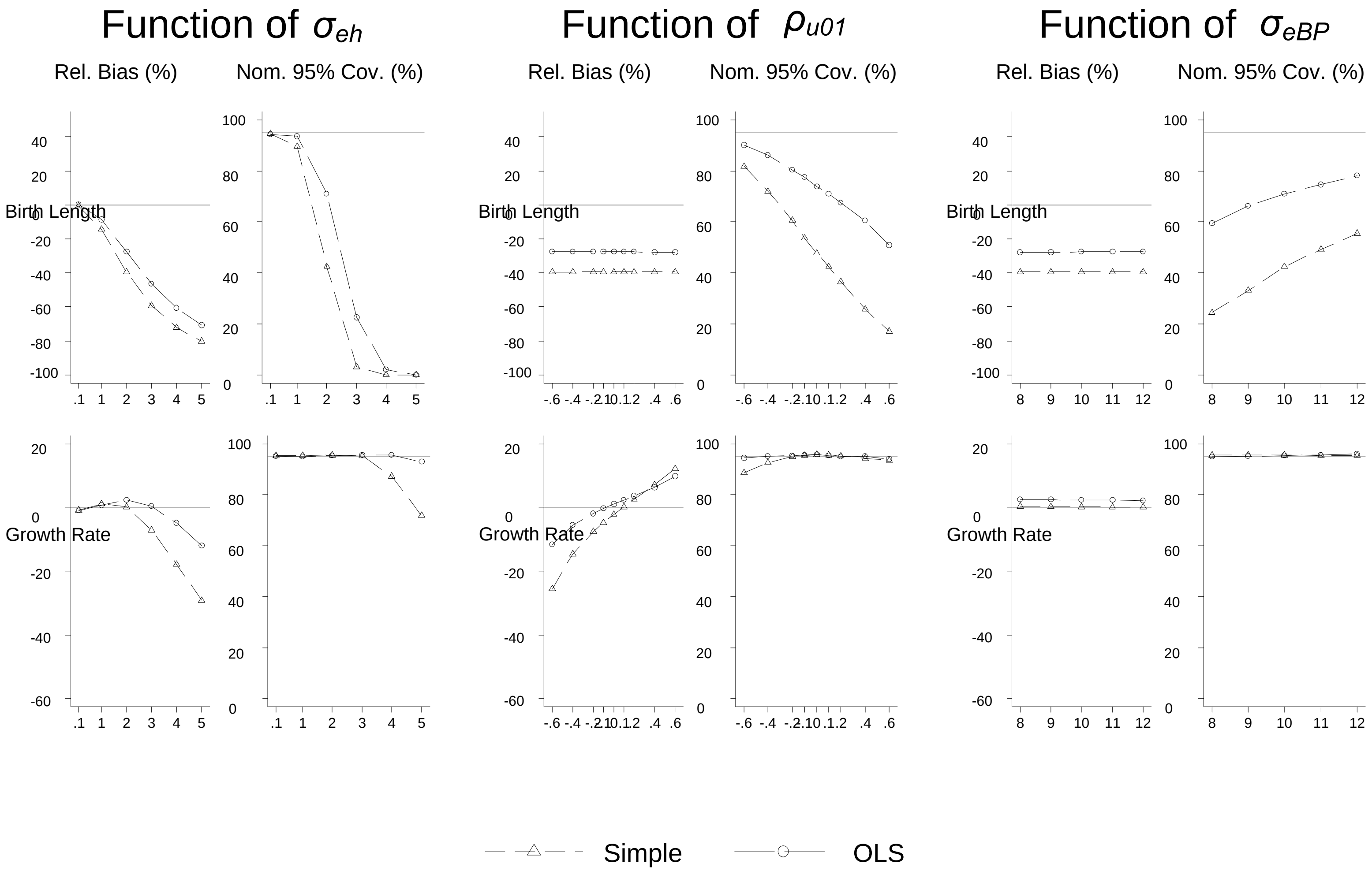

Supplementary Figure 2:

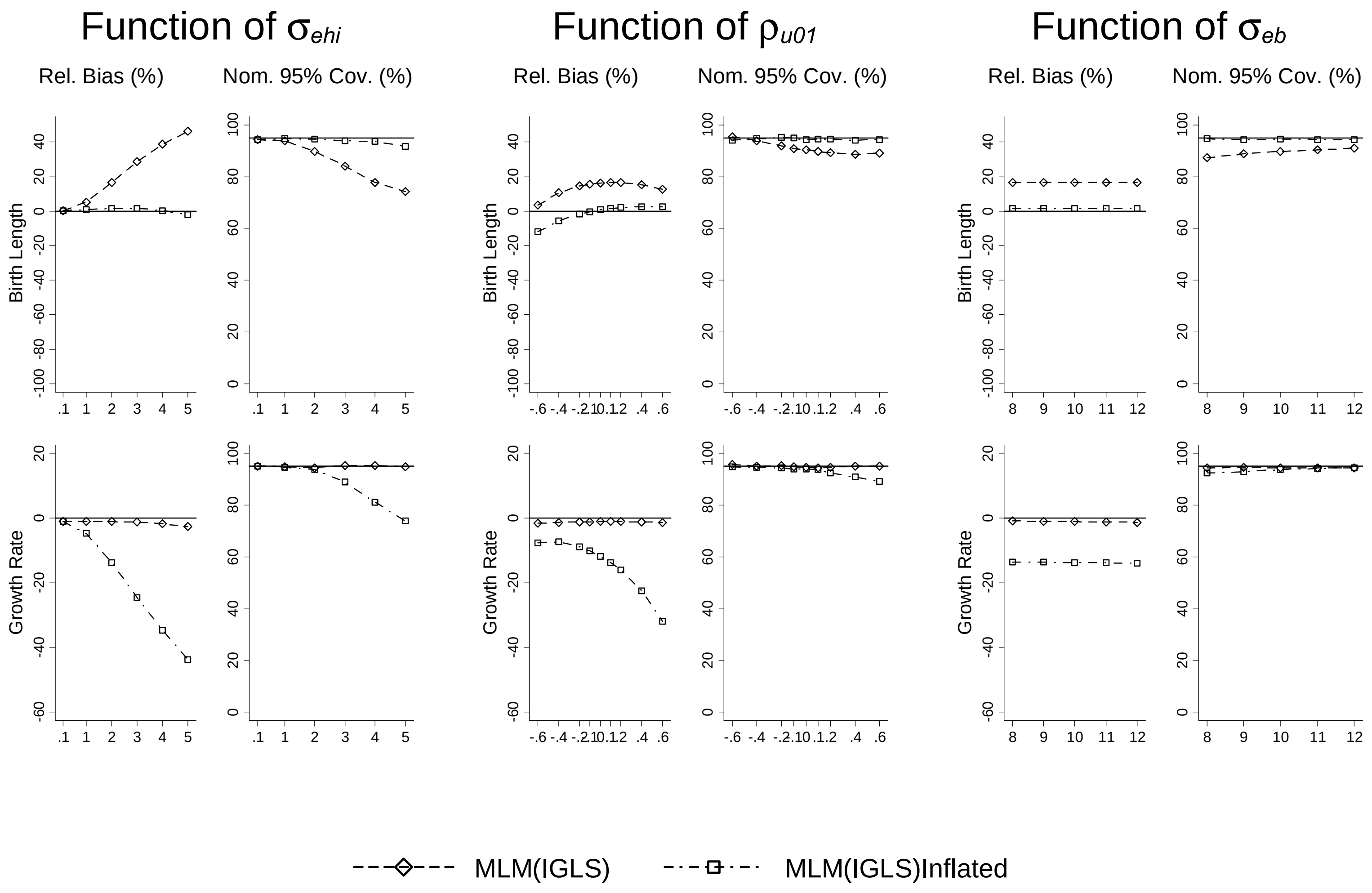

Supplementary Figure 3:

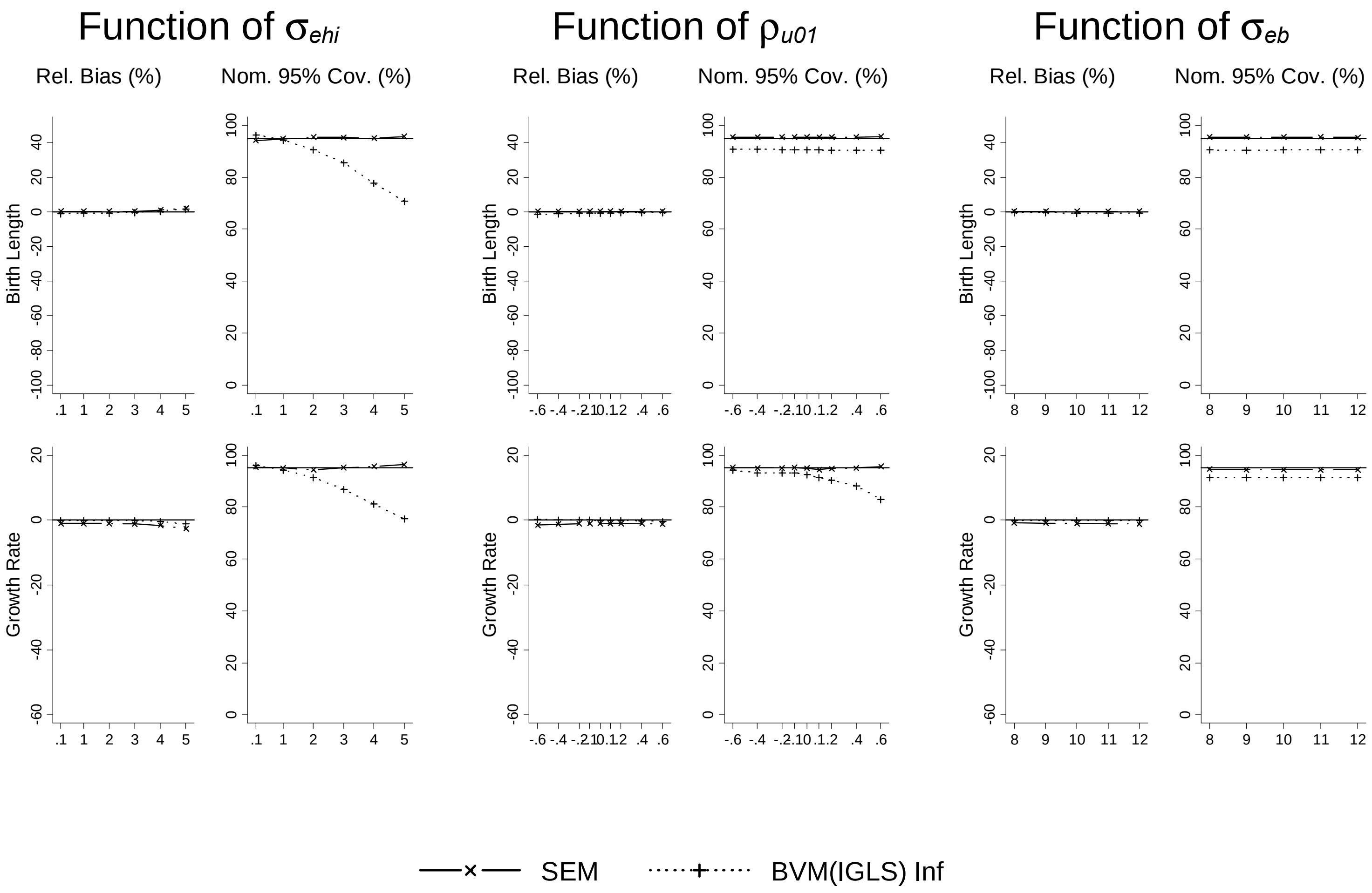

Supplement: Supplementary material [file SMM548822_supplementary_figures.pdf]
